# Ecology, Behaviour and Control of *Apis cerana* with a Focus on Relevance to the Australian Incursion

**DOI:** 10.3390/insects4040558

**Published:** 2013-10-21

**Authors:** Anna H. Koetz

**Affiliations:** Biosecurity Queensland, Department of Agriculture, Fisheries and Forestry, 21-23 Redden St., Portsmith, QLD 4870, Australia; E-Mail: ahkoetz@gmail.com; Tel.: +61-419-726-698; Fax: +61-7-4057-3690

**Keywords:** *Apis cerana*, *Apis mellifera*, incursion, pest species, Australia, pollination, competition, distribution, control

## Abstract

*Apis cerana* Fabricius is endemic to most of Asia, where it has been used for honey production and pollination services for thousands of years. Since the 1980s, *A. cerana* has been introduced to areas outside its natural range (namely New Guinea, the Solomon Islands, and Australia), which sparked fears that it may become a pest species that could compete with, and negatively affect, native Australian fauna and flora, as well as commercially kept *A. mellifera* and commercial crops. This literature review is a response to these concerns and reviews what is known about the ecology and behaviour of *A. cerana*. Differences between temperate and tropical strains of *A. cerana* are reviewed, as are *A. cerana* pollination, competition between *A. cerana* and *A. mellifera*, and the impact and control strategies of introduced *A. cerana*, with a particular focus on gaps of current knowledge.

## 1. Introduction

*Apis cerana* Fabricius (also known as the Asian honeybee, Asiatic bee, Asian hive bee, Indian honeybee, Indian bee, Chinese bee, Mee bee, Eastern honeybee, and Fly Bee) is endemic to most of Asia where it has been used for honey production and pollination services for thousands of years. *A. cerana* has been described as the exact equivalent of its European/African sister species *A. mellifera*, the European honeybee, showing as wide a range and capacity for variation and adaptation [1]. Similar to *A. mellifera*, *A. cerana* occupies a large range with varied climatic conditions, from cool regions in higher latitudes and altitudes, to dry, semi-desert environments, as well as tropical climates [1]. And similar to *A. mellifera*, *A. cerana* is also genetically and morphologically subdivided into several strains that differ in their ecology and behaviour, particularly between temperate and tropical strains.

However, following an intentional introduction of hives into West Papua in the 1980s, *A. cerana* has spread to areas outside its natural range (namely New Guinea, the Solomon Islands, and Australia), and the fear is that it may become a pest species that could compete with native Australian fauna (especially insects) and affect pollination of native flora. It is also feared that it may compete with, and rob the hives of, *A. mellifera*, which is commercially used for honey production and crop pollination services. Substantial impact of introduced *A. cerana* on *A. mellifera* hives have indeed been reported in the Solomon Islands [2,3,4], raising concern that *A. cerana* may have the same devastating effect on *A. mellifera*, and, consequently, on honey production and pollination services, in Australia. 

This literature review is a response to these concerns and aims to review the scientific literature to determine what is known about the ecology, behaviour, and control of *A. cerana*. The specific strain that was introduced to Australia is thought to be *A. cerana* Java genotype [5], *i.e.*, from the recognised Indo-Malayan strain of *A. cerana*, a tropical strain of the species found in Indonesia, Malaysia, Borneo, and Sulawesi. Although the literature review aims to clarify the ecology and behaviour of this tropical, Indo-Malayan strain in particular, very little is known and published about it. Therefore, the literature search was widened to *A. cerana* in general, with a focus (where possible) on tropical *A. cerana.* However, it is important to note that although *A. cerana* may behave in a certain way where it is endemic, one should not assume that a species will behave in the same way outside its natural range. Similarly, differences in behaviour may be found between different strains, and one should not assume that different strains will behave in the same way.

Specifically, this review aims to answer the following questions:

1. What is known about the ecology and behaviour of Indo-Malayan *Apis cerana*? How are tropical and temperate strains different?
2. What is known about honey production and pollination services in *A. cerana*? How does this differ between tropical and temperate strains?
3. How does *A. cerana* ecology and behaviour compare to *Apis mellifera*, and what is the likely ecological overlap between these two species where they co-occur?
4. What has been the impact of *A. cerana* where it has been introduced? How is *A. cerana* controlled and managed where it has been introduced?

This literature review is divided into several sections, covering the distribution of *A. cerana*, its ecology and behaviour, pollination, *A. cerana vs. A. mellifera*, and control strategies in place in Australia and elsewhere. In doing so, gaps in knowledge will be identified and recommendations for future research made.

## 2. Distribution

### Natural and Introduced Range

**Figure 1:**
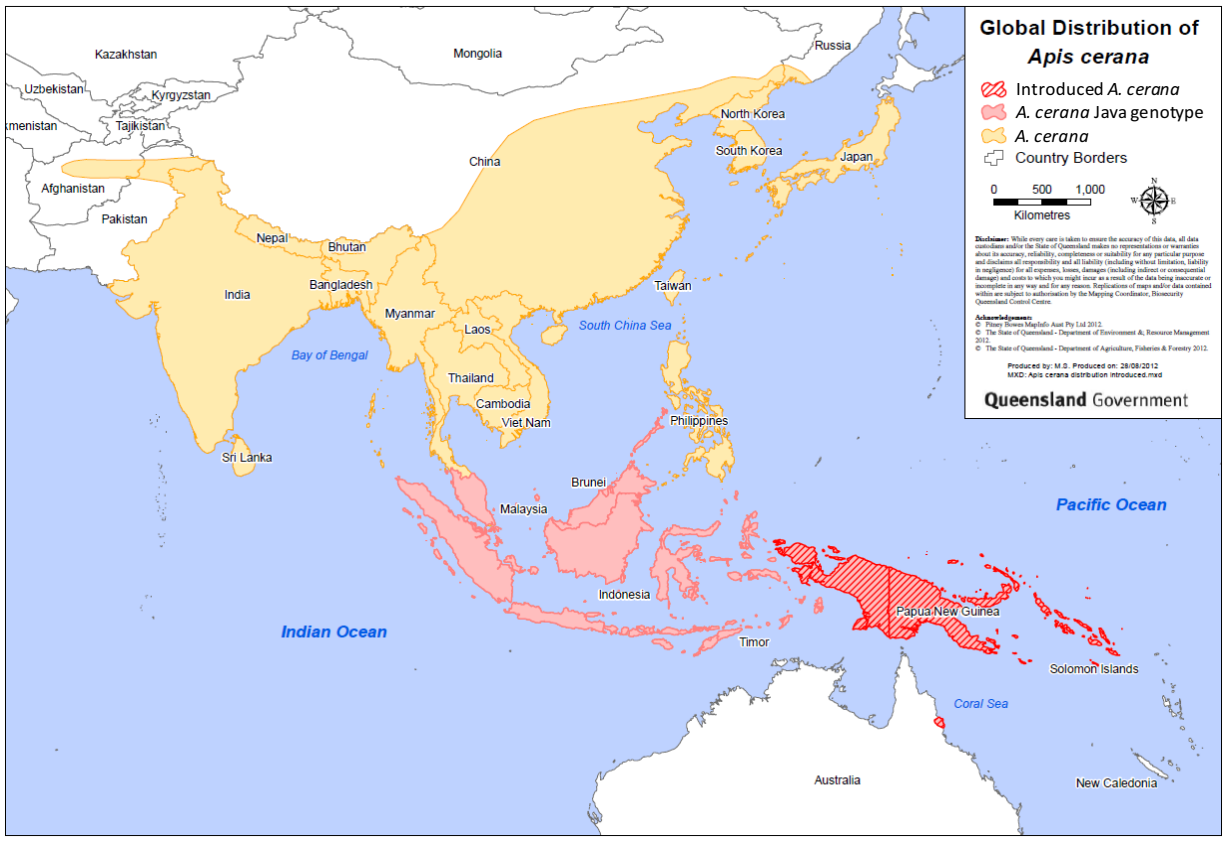
Distribution map of *Apis cerana* showing the species’ natural range (yellow and red) and its introduced range (stippled red).

The natural range of *A. cerana* is widespread across temperate and tropical Asia, reaching from Afghanistan to Japan, north into the foothills of the Himalayas, and south through Indonesia (Figure 1) [1,6,7]. *A. cerana*’s range covers many climatic zones, from tropical rainforest and tropical savannah to mid-latitude grasslands, moist continental deciduous forests to taiga [6].

*Apis* are not native to the Americas, Australia, New Zealand, or Pacific islands [8]. However, *A. mellifera* has been introduced to all of these areas, and *A. cerana* has been introduced to some.

*A. cerana* was intentionally introduced from Java into the Indonesian province of Papua New Guinea in the late 1970s. It then established throughout New Guinea (Figure 1) [9]. In 1993, swarms of *A. cerana* were detected on Boigu, Saibai, and Dauan islands in the Torres Strait [10]. *A. cerana* has been intercepted and destroyed on vessels at Australian seaports since 1995, namely Cairns, Brisbane, Melbourne, and Adelaide [11]. A nest was found in Darwin in 1998—it was destroyed and an eradication and surveillance program established [2]. In 2003, *A. cerana* was detected over 1,000 kilometres further east on the Solomon Islands (Figure 1) [2,3]. In 2007, a nest was found in Cairns, Australia [11,12]. Although this nest was destroyed, over 800 nests and swarms have been detected and destroyed since. In 2011, the eradication of *A. cerana* in Cairns was deemed not feasible by the National Management Group (formed by the Australian Government specifically to deal with *A. cerana* in Australia), and so an *A. cerana* population is now established in the Cairns region.

Other, less well documented, *A. cerana* incursions have also occurred in New Zealand and possibly Hawaii (mentioned in [1]). No further documentation of either incursion could be found in the scientific or common literature.

## 3. Morphology and Genetic Diversity

Not surprisingly, given its wide range covering many climatic zones, considerable genetic and morphological variation has been shown within *A. cerana* [1,13,14]. In order to place the *A. cerana* introduced to Australia within geographic and genetic context, this variation and the subgroupings or strains suggested for *A. cerana* will be summarised*.* It needs to be noted that there has been extensive debate, re-classification, and re-naming of *A. cerana* subgroups since the species was first described by Fabricius in 1793. Recent use of more sophisticated morphological and genetic techniques have started to shed light onto the evolution of *A. cerana*.

### 3.1. Appearance

There are nine currently recognised *Apis* honeybee species worldwide, eight of which are native to Asia. *A. mellifera* is the only *Apis* honeybee species outside of Asia. Among the Asian honeybee species, *A. cerana* is a medium sized honeybee—smaller than the giant Asian honeybees (*A. dorsata* and *A. laboriosa*) but larger than the dwarf Asian honeybees (*A.* *florea* and *A. andreniformes*) [15]. *A. cerana* is the smallest of the four cavity-nesting Asian honeybees (including *A. koschevnikovi*, *A. nuluensis*, *A. nigrocincta* and *A. cerana*) [15]. 

*A. cerana* are generally smaller than *A. mellifera* [15]. However, remarkable morphological variation has been found within both *A. cerana* and *A. mellifera*, with non-tropical bees being larger than tropical bees, and bees at high altitude being larger than those at low altitude [1,16,17]. Although *A. cerana* appear to be smaller in general, there is some overlap between larger, cool-climate *A. cerana* and smaller, warm-climate (African) *A. mellifera* [1]. In Queensland, Australia, *A. cerana* tend to be smaller than *A. mellifera* [18] but this has yet to be quantified. 

*A. cerana* have more prominent and consistent striping on their abdomen with even black bands across the entire abdomen, whereas *A. mellifera* tend to have uneven black stripes with thinner stripes at the front of the abdomen and thicker black stripes towards the back of the abdomen (making it appear more yellow at the front and darker at the back). However, colouration is notoriously variable in nature, and the most reliable morphological characteristic that distinguishes *A. cerana* from *A. mellifera* is the extension of the radial vein on the hind wing, which is absent in *A. mellifera* [1].

### 3.2. Morphological Divergence

*A. cerana* is morphologically and genetically subdivided across its range. Most recent studies found that there are six “morphoclusters”, *i.e.*, groupings within *A. cerana* based on complex statistical analyses of 12 morphological characters [13]. The genetic strain of *A. cerana* found in Australia, New Guinea, and the Solomon islands [5] falls within morphocluster VI, which is distributed across southern Thailand, Malaysia, and Indonesia (Indo-Malayan *A. cerana*) [13]. Morphoclusters V and VI (Philippine and Indo-Malayan clusters, respectively) also occur in tropical wet climate. All other morphoclusters occur outside wet tropical climates, although some subclusters may fall within wet/dry tropical or subtropical climates (within morphocluster I: Indus, Central and Eastern China, and Japonica subclusters; within morphocluster IV: Thailand subcluster).

**Figure 2:**
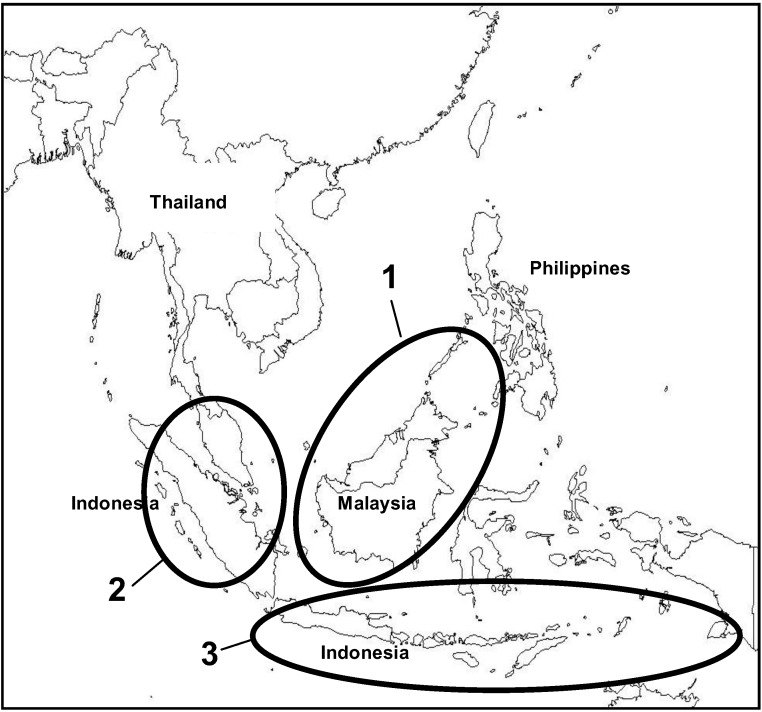
Subgroupings found within morphocluster VI, the Indo-Malayan *Apis cerana* according to [13]. (**1**) Palawan (Philippines), North Borneo (Malaysia), and Kalimantan (Indonesia); (**2**) Malay Peninsula, Sumatra, and some Sulawesi; (**3**) Indonesia (Java, Bali, Irian Jaya, some Sulawesi and Sumatera).

Subtle morphological differentiation has been detected within some of the morphoclusters, which is generally linked to biogeographical and climatic boundaries [13]. Within the Indo-Malayan morphogroup VI (containing *A. cerana* Java genotype), three main subgroups were found [13,19]: (1) Palawan (Philippines), North Borneo (Malaysia), and Kalimantan (Indonesia); (2) Malay Peninsula, Sumatra, and some Sulawesi; (3) Indonesia (Java, Bali, Irian Jaya, some Sulawesi and Sumatera (Figure 2).

### 3.3. Genetic Divergence

**Figure 3:**
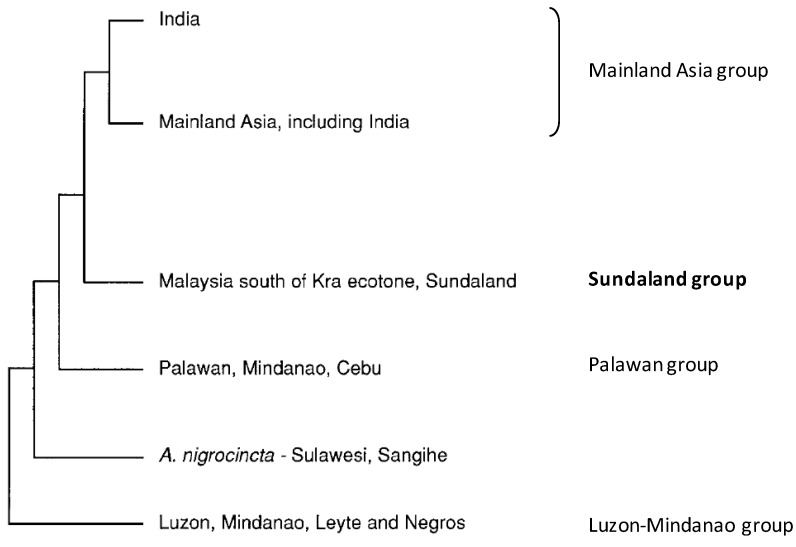
Phylogenetic tree of the main *A. cerana* haplotypes (mitochondrial DNA subgroupings) and their corresponding geographic regions (sourced from [14]). In addition, shown is the placement of *A. nigrocincta* within the tree.

Most recent genetic studies generally agree with the morphological studies. They divide the species into four main genetic groups (Figure 3) [14,20]. One of these groups (the Sundaland group) corresponds with morphogroup VI (=Indo-Malayan *A. cerana*) (Figure 3). This genetically and morphologically distinct subgroup is confined to the Asian tropics south of 10 °N latitude [14,20,21,22].

Further genetic subdivision can be found within the Sundaland/Indo-Malayan group. Relevant here is the fact that *A. cerana* samples from Java, Bali, Flores, Timor, and Sulawesi cluster together, as do samples from Bali and Lombok [14,20]. Genetic clustering within the Sundaland/Indo-Malayan group seems to be linked to location upon the Sunda continental shelf and sea level fluctuations during Pleistocene glaciations. Islands on the Sunda shelf (Sumatra, Java, Bali, Lombok, Timor, and Flores) would have been connected by dry land during glaciations periods, whereas Borneo and Sulawesi remained separated by deep channels [20].

Sharp genetic boundaries between populations (e.g., between the Mainland Asia group and the Sundaland/Indo-Malayan group) are linked specifically to the genetic marker used. Mitochondrial DNA is maternally inherited, and so any gene flow and admixture between populations represents female gene flow (migration), whereas drone gene flow is not detected using this marker. Differences in the nuclear genome, which evolves much more slowly and is both maternally and paternally inherited, would be much less than those observed in the above studies [20]. In addition, mitochondrial DNA gives a good picture of changes in population structure, while it gives little or no information about adaptation to local environments [20]. This means that differentiation within the Sundaland/Indo-Malayan group (as, indeed, between and within other morphogroups) is very slight (*i.e.*, differentiation does not warrant sub-species status [13,20]), and broad habitat differences rather than genetic differences may explain differences in behaviour.

The incursions into PNG and Australia clustered genetically with *A. cerana* of the Indo-Malayan region using mitochondrial DNA (CO I gene; [5]). A recent genetic study using more sensitive microsatellite genetic markers revealed that Australian *A. cerana* appear distinct from *A. cerana* in the Torres Strait and the Solomon Islands, as well as from *A. cerana* from Thailand [23]. However, sample sizes were limited and did not include samples from the Indo-Malayan group.

## 4. Ecology and Behaviour

### Foraging

Honeybees collect nectar and pollen, which are needed for bee nutrition. Pollen is a source of protein, nectar is a source of carbohydrates, and together they provide all the food necessary for larval growth and metamorphosis, and for adult function and development [24]. While bees collect nectar and pollen, they provide one of the most important ecological services—pollination. Pollination will be covered in more detail further on. Here, general foraging and its importance to the bee colony will be covered.

On a single foraging trip, *A. cerana* foragers tend to collect either pollen or nectar (not both) from a single species of plant, continuing to collect pollen or nectar from that plant throughout the day [25,26].

Foraging ranges of *A. cerana* vary between different studies, but generally *Apis* honeybees prefer to forage within 200–300 m of their nest [27]. In all studies reviewed here, half of the observations showed *A. cerana* foraging within 250 m from the hive, and most (95%) *A. cerana* foraged within 500–900 m (Punchihewa, 1985 in [25,28,29,30,31,32]). Maximum foraging ranges of 1,500 m to 2,500 m have been observed [28,31,33].

In comparison, *A. mellifera* tends to forage across much larger distances, with maximum distances of over 10 km [34,35,36]. Half of all foragers were found within 1,650 m, and most foragers (95%) were found within 6 km in a natural deciduous forest in North-Eastern US [34].

The time of day when honeybees start and finish foraging often depends on ambient temperature, humidity and/or light levels, as well as the availability of floral resources—the specific combination of factors is species-specific (reviewed in [36]). In general, *A. cerana* tend to start foraging earlier in the day than *A. mellifera* [25,32,37,38], as *A. cerana* require slightly lower temperatures, light intensity and solar radiation levels to commence flight activity than *A. mellifera* [37].

In an apple orchard in Northern India, *A. cerana indica* foraged earlier in the day compared to *A. mellifera*, being most active between 9 a.m. and 11.30 a.m. when temperatures were between 15.5 and 21 °C, compared to *A. mellifera*, which were most active between 11 a.m. and 1:30 p.m. at temperatures between 21–25 °C (Verma, 1995 in [25,38]). In Kathmandu, Nepal, there were two peak foraging times per day, which corresponded with temperatures of 20–21 °C [39].

Subtropical *A. cerana* in India also tended to start foraging earlier and finish slightly later than *A. mellifera*. However, the single peak foraging times seemed to overlap between the two species [37]. 

No information on foraging times could be found for indo-Malayan *A. cerana* in its native range or in Australia. Determining foraging times and peaks for both *A. cerana* and *A. mellifera* in Australia could provide some indication of potential resource competition between the species. One could hypothesize that *A. cerana* forages earlier than *A. mellifera* as elsewhere in its range, with one foraging peak in the morning, similar to subtropical India.

## 5. Nesting

### 5.1. Nesting Habitats and Sites

Four of the eight Asian honeybee species nest in cavities, including *A. cerana* [6,15]. In areas where *A. cerana* is the only cavity nesting species, it can be found nesting in all habitats, including primary forests [40,41]. Where *A. cerana* is found co-occurring with other cavity-nesting *Apis* species, it tends to prefer nesting in secondary forest, agricultural or disturbed areas (reviewed in [25,42,43,44]). In Central Sulawesi, where *A. cerana* co-occurs with the cavity-nesting *A. nigrocincta*, *A. cerana* has only been found nesting in agricultural areas and villages [25,44]. However, in North-Eastern Thailand where *A. cerana* is the only cavity-nesting *Apis* species, *A. cerana* were found nesting in a variety of habitats, predominantly in primary and secondary rainforest as well as cleared areas [40]. *A. cerana* is also found in the rainforests of Western Ghats and Sri Lanka [41]. Furthermore, in a review of beekeeping history and practices, mention is made of Javanese beekeepers taking log-hives into the jungle to catch swarms [7]. 

The majority of detections of *A. cerana* in Australia were made in open and disturbed habitats [29]. However, this does not indicate a preference for such habitats, but rather is likely to be due to substantially greater search effort in residential, industrial, and agricultural areas compared to difficult-to-search habitats (such as mangroves, rainforest, and Eucalypt woodland). Indeed, a number of nest detections in mangroves, rainforest and Eucalypt woodland [18] confirms that *A. cerana* can and does exploit these habitats in Australia.

*A. cerana* nests are generally found in tree hollows, rock crevices, caves, and house cavities. In Padang, Sumatra, as well as in Bangladesh, *A. cerana* mostly nested in tree hollows [45,46], whereas in Thailand, the majority nested in caves [40]. In West Pakistan, wild *A. cerana* were mainly found in tree hollows or rock crevices [47], but also cavities in house walls [48]. Hollow trunks of coconut palms as well as piles of coconut husks seem to be a preferred nesting site in areas where coconut plantations abound [49]. In one study, an *A. cerana* nest was found in the ground [46]. In Queensland, Australia, *A. cerana* nests have been found equally in human and natural structures, including, for example, wall and roof cavities, garden sheds, compost bins, letter boxes, vehicles and machinery, as well as trees cavities [29].

In its natural range, *A. cerana* tend to nest at relatively low heights, with average nest heights of one to two metres [40,46] and maximum nest heights varying between two and ten metres depending on the study [40,45,46,49,50]. *A. cerana* in Queensland, Australia, have been found nesting at a much higher average height of 4.45 m and up to 30 m (N = 327; [29]). It is unknown whether this difference is due to different nesting behaviour in Australia, or whether nest height data may be generally biased due to the difficulty of finding nests at large heights. 

Nest cavity volumes of *A. cerana* vary greatly, from 2.75 to 110 litres [45,46,49]. Due to this variation, averages also varied between studies and need to be regarded with caution—from 10–15 litres in general [42] to 23.5 litres and 45.9 litres in West Sumatra, Indonesia [45,49]. The smallest and largest cavity volumes found in the literature were found in West Sumatra, Indonesia [45,49], the home of Indo-Malayan *A. cerana*.

In comparison, *A. mellifera* nest cavity volumes have been found to vary between 12 and 443 litres with most being approximately 35 litres [51] and a preference for cavities between 20 and 80 litres [52].

It is thought that introduced *Apis* species, including *A. cerana*, may compete with Australian native fauna such as birds, bees, and mammals for nesting sites. Given the overlap of nesting sites and nest cavity volumes, it is conceivable that feral colonies of *A. cerana* and *A. mellifera* may compete for nesting sites. Similarly, it is possible that any introduced *Apis* species may compete with native Australian fauna for nesting sites. However, results are as yet inconclusive [53,54,55,56,57,58,59,60,61,62].

### 5.2. Nest Characteristics

*A. cerana* build multiple comb nests in dark cavities [42], although open nests (e.g., built underneath building eaves) have also been observed ([18]; Oldroyd, pers. obs). Combs are built parallel with a uniform distance between them (the bee space; [42]). Honey is stored in the upper part of the combs as well as in the outer combs adjacent to the cavity walls; the remaining comb space is taken up by brood of various ages [42,63].

The number of combs in *A. cerana* nests varied from three to fourteen combs (Lavrekhin, 1958 in [1,40,45,46]) with an average of 6.4 in Bangladesh [46], 5.6 in Thailand [40], and 7.9 in West Sumatra, Indonesia [45].

*A. cerana* cells are of two sizes: smaller worker cells (diameter of 3.6–4.9 mm, depth of 1.01 mm; Tingek, 1996 in [1,42,45,46]) and larger drone cells (diameter of 4.7–5.3 mm; [1,42,46]). In comparison, *A. mellifera* worker cell sizes were approximately 4.9 mm average [64]. *A. cerana* drone cells have a distinctly raised cap with a unique pore at their apex [42,65]. The size difference between worker cells and drone cells is less pronounced in *A. cerana* than in *A. mellifera* [1]. Queen cells are large conical cells built on the lower edge of the combs [42]. However, just as body size varies geographically, so does worker cell size. Worker cells are larger in colder regions (e.g., Japan: 4.7–4.8 mm, High Himalaya: 4.9 mm, Central India: 4.5 mm, Southern India: 4.3 mm, Phillippines: 3.6–4.0 mm; Crane, 1993 in [1,42].

Inoue *et al.* [45] reported in great detail nest site and nest characteristics of 10 *A. cerana* nests in West Sumatra (reported as *A. cerana indica* but more likely to be Indo-Malayan *A. cerana* according to current classification). Nests had on average 7.9 (3–14, SE ± 3.9) combs, and combs were on average 51.6 ± 21.6 cm high and 18.2 ± 7.1 cm wide with a volume of 22.3 litres and a weight of 1.7 kg. Average number of cells was 28,352 (5,315–69,515) [45].

### 5.3. Colony Size and Nest Density

Reported total colony sizes (number of bees/colony) vary greatly in *A. cerana*, ranging from 1,400–2,000 bees when lacking in cavities of sufficient size [1], and up to 34,000 bees [45]. Average sizes vary also, from 6,884 to 14,745 bees (wild colonies; [40,45,46,66]) and 13,164 bees (hived colonies; [67]). In Australia, rather small average nest sizes of 2,182 bees (wild colonies; 41 up to 10,706 bees) were reported [29]. In comparison, *A. mellifera* colony sizes have been reported between 15,000 bees [67] and up to 50,000 bees (reviewed in [68]).

There are few studies that estimate nest density in *Apis* species in general [69,70], and in *A. cerana* in particular. One single study on *A. cerana* nest density found it to be 22 nests/km^2^ in Padang, Sumatra, with a mean distance between nests of 104 metres (67–244 m, SE ± 36; [45]). It is unknown whether density saturation had been reached. No information on nest densities in Australia is currently available.

*A. mellifera* densities vary greatly and have been estimated from <1 nest/km^2^ in cooler climates such as Northern Europe, Russia, and North-Eastern USA (Galton, 1971 in [15,50,70,71]), to 107 nests/km^2^ in a dry tropical forest in Brazil (Michener, 1975 in [69]). *A. mellifera* density in a Ugandan tropical forest reserve was 12 nests/km^2^ [69]. Climate was found to be correlated with colony densities [70], and so larger nest densities (>9) were found in warmer climates across Europe, Africa, and Asia [70,71].

In South Australia, *A. mellifera* densities were found to vary greatly, from 0.1 nests/km^2^ (mallee heath) to 10–40 nests/km^2^ (Eucalypt woodland), with localised densities of 1,000 nests/km^2^ [72]. Oldroyd *et al.* [56,73] reported densities from 50 to 150 nests/km^2^ in Wyperfield National Park, Victoria. However, their estimates were restricted to a narrow strip of riparian woodland within the National Park [56]. When converting this to a density per square kilometre, the density was 7.7 nests/km^2^ [74]. A more recent, genetic study inferred *A. mellifera* nest densities of 4.4 to 27.7 nests/km^2^, with significantly higher densities in undisturbed habitat [75].

## 6. Mating and Reproduction

As in all other *Apis* species, mating behaviour characteristics of *A. cerana* include: (1) large numbers of drones trying to mate with each queen (effective sex ratio during mating flights is highly skewed towards males), (2) drones dying shortly after mating, (3) queens mating with many drones on the mating flight, (4) drones and queens mating on the wing, and (5) drones aggregating at specific locations (drone congregation areas) (reviewed in [76]). 

The mating season of *Apis* species is inevitably linked to the swarming season and the seasonal blooming cycles. Because of this, in many areas, different *Apis* species reproduce simultaneously, which could lead to reproductive overlap [77]. Differences in the timing of mating flights, sex attractants (pheromones) and drone congregation areas are important in establishing and maintaining isolation between different *Apis* species [1,77]. However, there seems to be some overlap in all three aspects between *A. cerana* and *A. mellifera*.

### 6.1. Timing of Mating Flights, Sex Attractants, and Drone Congregation Areas

Different *Apis* species generally have distinctly different drone flight timings, and this characteristic has been used for recognition of new species [1,77]. However, flight timing shows great intraspecific variation and appears to be under great selective pressures, particularly in areas where several *Apis* species naturally overlap [78]. In areas of (natural) overlap, flight timings for each species were found to be shorter than in areas of non-overlap [78].

*A. cerana* and *A. mellifera* have been found to have very similar drone flight timings in Europe and Japan, generally occurring between noon and mid to late afternoon (3–5 p.m.) although the exact timings change between study locations [1,15,63,77,78]. No information is available on drone flight timing of Indo-Malayan *A. cerana* in the published literature. However, drones of both *A. cerana* and *A. mellifera* have been observed to fly between 1:00 p.m. and 5:30 p.m. in Cairns, Australia [79].

*Apis* drones are specifically attracted to 9-oxodec-trans-2-enoic acid (9-ODA) queen mandibular pheromone, which does not seem to be species-specific, leading to mating interference between species (reviewed in [77]).

Similar to other *Apis* species, *A. cerana* gather in well-defined drone congregation areas (DCA) that are perennial and can stay in the same location for up to 25 years (reviewed in [15]). Such DCAs facilitate rapid mating of young queens with many drones in each short queen mating flight (Woyke, 1975 in [76]). Locations of *A. cerana* DCAs vary between studies [77]. *A. cerana* drones were observed to gather close to trees and restrict their flight to the open space between the trees in Sri Lanka and Borneo [77,80]. In Japan, *A. cerana* drones were observed using prominent trees as landmarks where they assembled under the branches [81]. In Germany, *A. cerana indica* imported from Pakistan congregated in an open valley far from trees [47], similar to the open-air DCAs in *A. mellifera* [77]. No information is available on *A. cerana* drone congregation areas in Australia.

Such distinct overlap in drone flight timing, sex pheromones, and DCAs leads to mating interference between the species in some areas, where *A. cerana* are generally negatively affected by the presence of *A. mellifera* (see Section 10.4 on mating interference below). If mating interference also occurs in Australia, *A cerana* may not thrive and spread as it would in the absence of *A. mellifera*. 

### 6.2. Brood Development

*A. cerana* development is very similar to that of other *Apis* species in general, and that of *A. mellifera* in particular. *A. cerana* brood development is slightly faster than that of *A. mellifera*, except for *A. cerana* queens (Table 1) [15,82]. However, it seems unlikely that this slight difference will affect competition or invasiveness. As in other cavity-nesting species, the larva’s brood cell is capped by the workers just before the last of the five larval instars [15].

**Table 1:** Duration of the life cycle (days) of different castes of *A. cerana* and *A. mellifera* modified from [15].

Stage	Worker		Drone		Queen	
	*A. cerana*	*A. mellifera*	*A. cerana*	*A. mellifera*	*A. cerana*	*A. mellifera*
Egg to larva	3	3	3	3	3	3
Larva to pupa	5	6	6	7	4–5.5	5
Pupa to adult	11	12	14	14	6–7.5	5
Total	19	21	23	24	13–16	13

## 7. Swarming and Absconding

There are two types of swarming in all *Apis* species: reproductive swarming, and absconding. Reproductive swarming involves the splitting of a colony and movement of the old queen (with >70% of the colony) to a new nest site, while the new queen stays with the remaining colony and all its resources (honey, pollen, brood) in the old nest site. It generally occurs when conditions are favourable and floral resources are abundant e.g., [67].

There are two types of absconding: seasonal absconding or migration, which is the movement of a whole colony due to resource depletion, declining nest site quality, and/or chronic disturbance; and disturbance-absconding caused by acute disturbance (natural, e.g., fire, flooding; or anthropogenic, e.g., intervention by beekeepers) [6]. Seasonal absconding involves a period of time preparing for the move (lasting days to weeks) prior to moving, when foraging, honey and brood levels are reduced. No such preparation occurs before disturbance absconding.

In general, tropical honeybee species (*Apis*), including African strains of *A. mellifera*, are more prone to absconding than temperate species due to the fact that environmental conditions (temperature, humidity and resource levels) are more favourable for survival year-round. This means that, unlike temperate honeybees, tropical honeybees are able to move the whole colony throughout the year in response to change or disturbance, and to follow the honey flow [83], both of which increase fitness and survival [1,68]. In contrast, temperate honeybees had to evolve in conditions that are favourable only during a short period of time with long periods of food shortage and freezing temperatures, leading to hoarding of large honey stores and “staying put” in thermally stable nests in order to survive the unfavourable conditions of winter (e.g., temperate *A. mellifera*) [1,50,83].

### 7.1. Seasonal Absconding

Seasonal absconding is strongly related to resource depletion and adverse environmental conditions in the current location. *A. cerana* do not store large amounts of honey, and so they do not have sufficient stores to last through a long period of unfavourable conditions [15]. Instead, they move to find better conditions elsewhere, and so they have been seen to move, for example, during periods of high temperatures, after abatement of prolonged heavy rains, and during the dry season (reviewed in [6]). In the mountainous Sichuan Province in China, temperate and tropical climates occur in close proximity depending on altitude, leading to flower blooms that are short and widely spread geographically (Chen 1995 in [6])—here, *A. cerana* migrate all year, following the flower blooms. Absconding has also been found highest in areas with high environmental uncertainty (e.g., drought; [84]), and when nest cavities are too small for the growing colony [84]. However, studies on *A. cerana* in Southern China, Sumatra and India have also observed absconding irrespective of colony size, congestion or food supply (reviewed in [85]), or without an apparent external cause [1]. *A. cerana* in temperate areas seem to abscond less than those in tropical areas. For example, in Kashmir, Northern India, absconding is less likely, whereas in Thailand, all colonies absconded after the honey harvest (Akranatakul, 1984 in [1]).

*A. cerana* preparing for migration (seasonal absconding) are characterised by decreasing numbers of pollen-carrying workers, greatly reduced brood feeding and rearing, and reduced predator and parasite defence [6,15]. In addition, honey and pollen stores, eggs, and open and closed brood decrease dramatically, leading to large changes in colony demography [80,86,87].

*A. cerana* abscond less often than open-nesting Asian honeybee species [15] but much more often than temperate *A. mellifera*. Temperate *A. mellifera*, especially wild colonies, may abscond for to the same reasons as tropical honeybees do—depleting resources and starvation, predation, disturbance, adverse environmental conditions, and disease/parasitism [1].

The only information on absconding in Indo-Malayan *A. cerana* includes observations by Biosecurity Queensland DAFF operations staff who reported a nest absconding when attacked by green ants, and another after strong disturbance by humans [29].

Based purely on the higher levels of absconding in *A. cerana* compared to those of the temperate strains of *A. mellifera* found in Australia, *A. cerana* is likely to be (1) more invasive, spreading more frequently and at lower levels of disturbance; and (2) more competitive in terms of exploring and exploiting new habitats. However, other factors need to be taken into account, e.g., competitive ability. This will be discussed further on.

### 7.2. Predation (Disturbance Absconding)

Tropical honeybee species seem to be under more severe predation pressure than temperate honeybee species. Predation is thought to be an important and powerful force in the evolution of Asian honeybees, shaping choice of nest site, nest architecture, population size, worker morphology, and behaviour [40,50]. In a study on three co-occurring honeybee species in a semi-evergreen rainforest in North-East Thailand, each month, 10% of all observed *A. cerana* nests were forced to abandon their nest due to predation [40,50]. One *A. cerana* nest was destroyed every 10 months [40,50]. 

Natural predators of honeybees are attracted to all parts of the colony, including adult bees, larvae and pupae, pollen, honey, and wax [88]. Natural predators of *A. cerana* include wasps and hornets [15,47], which tend to prey on foragers but also at times attack colonies [15]. Ants also attack *A. cerana* colonies, including Green ants (*Oecophylla smaragdina*) and smaller ant species [15]. Vertebrate predators of *A. cerana* include toads, frogs, lizards and geckos, rats, honey badgers, macaque monkeys, tree shrews, most Asian bears (such as Malayan honey bear, Sloth bear, and Asiatic black bear), martens, tigers, and many birds (e.g., honey buzzards, bee-eaters, swifts, drongos, and honeyguides), as well as humans [15,88]. Amongst this list, the predators that also occur in Australia include ants (including Green ants), possibly some Australian native wasps and/or hornets, lizards, frogs and toads, Rainbow bee-eaters, swifts, and drongos, and, of course, humans. Of these, all but wasps, hornets, and drongos have been observed preying on *A. cerana* [40,50,89].

No information is available on the levels of predation pressure on *A. cerana* in Australia compared to those in its native range. Thus, no conclusions can be drawn whether predation in Australia may increase of decrease the species’ invasiveness.

In cavity-nesting *Apis* species, the main defence against predators is living in a protected cavity with a small entrance that can be easily guarded [15,88]. Colony defence behaviours include abdomen shaking, hissing (through wing vibrations), group defence (including grasping, pulling, and biting, as well as forming a “bee-ball” around wasps, killing by over-heating and/or asphyxiation), and stinging, which is the bees’ main defence against vertebrates [1,15,88,90].

### 7.3. Reproductive Swarming

Reproductive swarming in *A. mellifera* generally occurs when floral resources have been abundant, and a colony is performing well and outgrowing its hive space [63,68,77,91]. Little is known about swarming behaviour of *A. cerana*—most knowledge comes from *A. mellifera* [15]. From *A. mellifera,* it is known that soon after a swarm has left the old nest and settles tens of meters away, scouts will start searching for suitable nest sites. Similarly, *A. cerana* have been seen to settle 20–30 m away from the old nest, stay for several days, and then move to the new nest site [15]. An *A. cerana* swarm also tended to settle on a near-by tree after emerging from a hive in West Pakistan [47]. In a study on co-occurring *A. cerana* and *A. nigrocincta* in Sulawesi, Indonesia, dancing *A. cerana* scouts indicated distances of potential nest sites up to 1,420 m, but final distances that swarms actually travelled to their new nest site were found to be between 99 m and 780 m [25]. Nothing further is known about distances travelled to form a new nest.

Managed *A. mellifera* colonies are generally prevented from swarming by good colony management, removing new queen cells, re-queening and using queen excluders [92]. Wild, temperate *A. mellifera*, however, swarm nearly every year (and sometimes up to three times per seasonal cycle) in late spring or early summer when resources are highest [50,91]. When and how often *A. cerana* swarm is highly variable and depends on the geographic location and climate. *A. cerana* can swarm several times a year [1]. In Northern Pakistan, swarming will start once a colony reaches 20,000 bees, with an average of eight swarms per colony (Koeniger, 1976a in [1]). In western Pakistan, *A. cerana* was found to swarm twice a year, with up to 10 swarms produced per swarming season (average of six swarms) [47].

As reviewed in Hepburn [85], timing of swarming has been found to vary from no seasonal rhythm (Sumatra & Southern India), biphasic (Sumatra, Southern India, Pakistan, Japan & China; Vietnam, [67]), to distinct times of the year (Plains of India—April–May; Kashmir Valley—June–July; Pakistan—February/March and August/September, [47]). In Punjab, Northern India, most swarms issued before 1 p.m., with an average weight of 1 kg and a maximum weight of 1.8 kg (equalling approximately 16,000 bees [1,93]).

When foraging conditions are good over extended periods of time (as for example in the tropics), swarming will occur more frequently, and swarming and the production of queens and drones will be asynchronous. For example, Africanised honeybees (*A. mellifera scutellata*) swarmed up to 12 swarms per cycle (Winston, 1990 in [91]), and *A. cerana* produced up to eight swarms in Northern Pakistan (Koeniger, 1976 in [91]). When foraging conditions are good only at certain times of the year (e.g., spring and summer in temperate zones), swarming will occur during those specific times, and swarming and the production of queens and drones will be synchronous (as seen in temperate *A. mellifera*) [91].

Little is known about frequency and timing of swarming of *A. cerana* in Australia. Swarms were reported in any month of the year between 2009 and 2011 in Cairns, Australia [29]. No seasonal rhythm was apparent. However, it is unknown which of these swarms were reproductive and which were absconding swarms [29]. In Australia, swarms had an average size of 2,676 bees (466–6,800 bees; N = 65) [29], which appears much smaller than reported in the literature for within the natural range of *A. cerana* [67].

## 8. Other Behaviour

### 8.1. Temperament

*A. cerana* has been described as docile [33], mild [30], tolerant and timid [1], with a gentle temperament (Verma, 1990 in [27]) and low stinging tendency [1], although it will sting when cornered or highly disturbed. *A. cerana* is said to be less prone to stinging than *A. mellifera* and has less alerting pheromone in its sting (half the amount of *A. mellifera ligustica*, the Italian bee)—resulting in fewer additional stings by defending bees [1]. In a simulated attack on their nest, *A. cerana* guards simply retreated into their nest cavity [1,50]. When destroying nests as part of the control measures in Australia using aerosol spray in close proximity to the nest, bees rarely sting [18].

### 8.2. Diseases and Hygiene

Where honeybee species coexist they are bound to interact in some way (e.g., robbing) and so parasites and pathogens can be transmitted between species [94]. This is particularly worrisome where species that would not naturally come into contact coexist (e.g., *A. mellifera* and *A. cerana*) [94,95]. Diseases and parasites have been introduced from exotic *A. mellifera* to native *A. cerana* and *vice versa* (e.g., *Varroa destructor* from *A. cerana* to *A. mellifera*, and the tracheal mite *Acarapis woodi* as well as Israeli acute paralysis virus from *A. mellifera* to *A. cerana*) [94,96]. 

*A. cerana* diseases in Asia include bacterial infections (American and European foulbrood), protozoan and fungal infections (*Nosema ceranae* and *N. apis*; and chalkbrood), and virus infections (*Apis* Iridescent virus, Deformed wing virus, Kashmir bee virus, Thai sacbrood virus, black queen cell virus, Israeli acute paralysis virus) [15,68,94,97,98,99,100,101,102]. *A. cerana* parasites include *Varroa* (*V. destructor*, *V. jacobsoni*, and *V. underwoodi*) and tracheal mites (*Acarapis woodi*), as well as non-parasitic mites (reviewed in [15,68,103]). In addition, wax moth is also found in *A. cerana* (reviewed in [15]).

A recent study on the disease status of *A. cerana* in north-eatern Australia established the presence of chalkbrood and *Nosema ceranae*, as well as Black queen cell virus, Kashmir bee virus, Lake Sinai virus 1 and 2, and several, as yet, uncharacterised viruses [104]. In addition, all nests and swarms detected in the Cairns area in north-east Queensland, Australia, since 2007 have been checked for the presence of *Varroa* spp., tracheal mites, *Tropilaelaps* spp., and *Nosema* spp. No *Varroa*, *Tropilaelaps* or tracheal mites have so far been found in the Cairns population. However, *Nosema* was also found in some colonies [18].

*A. cerana* workers have been found to clean themselves and each other more thoroughly than *A. mellifera* [105,106,107,108]. In addition, infected brood is either removed before capping (e.g., larvae infected with American foulbrood or worker brood with *Varroa*) [97,108], or it is entombed (e.g., drone larvae infected with Varroa) [105,106,107]. Experiments also showed that the presence of *Varroa* semiochemical compounds result in immediate cleaning behaviour in *A. cerana* but not in *A. mellifera* [109,110].

*A. cerana* are generally regarded as much more hardy and disease-resistant than *A. mellifera*, making it a better species in many poorer areas of Asia as *A. cerana* requires less management and treatment for diseases [33,84,111].

### 8.3. Fanning Behaviour

One recognisable difference between *A. cerana* and *A. mellifera* is the fanning position of workers at the hive entrance—*A. cerana* workers ventilate the hive by fanning with their heads away from the entrance, whereas *A. mellifera* fan with their heads turned towards the entrance [1]. As entrances of hived bees are generally at the bottom of the nest/hive, this results in *A. cerana* workers facing upwards, whereas *A. mellifera* workers face downwards [1,33].

## 9. Pollination

One of the most important (but less obvious) services provided by bees is pollination. Pollination has two important consequences: it maintains biodiversity of flowering plants, and it maintains ecosystem function. Reduced pollination can lead to local extinction of plant species, a decline in fruit and seed-eating animals, loss of vegetation cover and, ultimately, the loss of a healthy ecosystem and its services. In agriculture, lack of adequate pollination can lead to deformed fruit and reduced crop yield [27].

Approximately 80% of all flowering plants depend on biotic pollinators [27], and an estimated 75% of the world’s crops benefit from biotic pollination [112]. About one-third of the global food production depends on biotic pollinators, particularly bees [113]. Bees also play an important role in tropical areas—social bees were found to pollinate 32% of plant species in lowland dipterocarp rainforest in Sarawak, Malaysia [114] and 72.7% of cultivated tropical plant species were pollinated by bees (21.3% by honeybees; Roubik 1995 and Nabhan & Buchmann 1997 in [115]).

Wild bees also play a vital role in pollination, particularly in Australia where farmers rely mostly on the free services of feral bees (*A. mellifera*) for pollination [116,117].

### 9.1. Pollination Services—Crops

In Asia, *A. cerana* is regarded as an excellent crop pollinator for a large variety of fruit and vegetable crops, sometimes outperforming *A. mellifera* [27,118,119,120,121,122,123,124]. This is thought to be due to the fact that *A. cerana* begin foraging earlier in the day and cease later in the day, pollinating flowers for longer than *A. mellifera*, and also because *A. cerana* employ relatively larger numbers of pollen collectors (compared to nectar collectors) than *A. mellifera* [27,118,119,120,121,122].

*A. cerana* has been reported as pollinating fruit and nuts, vegetables, pulses, oilseeds, spices, coffee, as well as fibre and forage crops, and has been found especially important in pollinating cauliflower, onion, and okra in India (reviewed in [27,112,123,125]). Studies specifically undertaken to show the impact of *A. cerana* on crop yield and productivity showed that pollination by *A. cerana* increased fruit and seed set, increased the quality of fruit and seeds, and reduced premature fruit drop (reviewed in [27,112,125]). Apple, peach, plum, citrus, and strawberry all showed a marked increase in fruit set (10 to 112% increase) and weight (33 to 48% increase). Similar results were also shown for a broad range of vegetables, oil rape seed, sunflower, buckwheat, soybean, cotton (reviewed in [27]), and coffee [125].

However, most of the studies reviewed in Partap [27] were conducted in temperate climates on temperate *A. cerana*. Few studies could be found on crop pollination of *A. cerana* Java genotype. One study on pollination of the non-food crop *Jatropha curcas* in Java, showed both *A. cerana* (presumably Java genotype) and *A. mellifera* to be pollinators [126]. Although *A. mellifera* seemed to be better pollinators than *A. cerana* for this particular crop, there was no statistical significance andsample sizes were very small [126].

These crops are also present in Australia and are likely to provide invading *A. cerana* with an abundant food resource. In addition, as, in Australia, these crops are pollinated by *A. mellifera*, there is potential for resource competition between the species. The potential for *A. cerana* to pollinate crops in Australia, and to potentially introduce competition for *A. mellifera* and/or native Australian bees, needs to be further explored.

### 9.2. Pollination Services—Wild Flora

*A. cerana* is an important canopy pollinator in the rainforests of Western Ghats and Sri Lanka, but little is known about the relationship between wild *A. cerana* and wild flora in other parts of Asia or the world (reviewed in [41]). At high altitudes in the Asian tropics, and in North-Eastern Asia, *A. cerana* is the only social bee present and so is likely to be an important if not the main pollinator (reviewed in [41]). For example, on the Amami Islands (300 km off the southernmost tip of Japan), *A. cerana* is the only bee pollinator during winter months (Kato, 2000 in [41]). In Hong Kong, *A. cerana* is a very important pollinator as it is the dominant visitor to 55% of the 83 woody plant species studied (Coreltt, 2001, in [41]). *A. cerana*’s ability to thrive in disturbed landscapes may also give it an important role as a pollinator, compensating for loss of other pollinators, similar to the role of *A. mellifera* in tropical America [41].

It is still unclear whether (and how) introduced honeybees impact on Australian native ecosystems. Research has found that some native flora are negatively impacted by honeybees (*A. mellifera*), some flora was positively impacted and some not at all [127,128,129]. It is also thought that *A. mellifera* is a less effective pollinator of Australian flora than native bees, may remove pollen without pollination occurring (pollen robbing), and that generally *A. mellifera* will cause changes to the abundance of native flora and fauna (reviewed in [60]). No studies have been conducted on the effect of *A. cerana* on the Australian native (see below). Furthermore, little is known about the role of *A. cerana* in pollinating and hence helping the spread of unwanted flora (weeds). However, it has been shown that, for example, the spread of *Phyla canescens* (Lippia) and *Cytisus scoparius* (Scotch Broom) in Australia is greatly facilitated by *A. mellifera*, as it is the primary floral visitor of these weeds that require insect pollination for successful seed set [130,131]. *A. cerana* may play a similar role and may facilitate the spread of some weeds.

## 10. *A. cerana vs. A. mellifera*

### 10.1. Competition between *A. cerana* and *A. mellifera*

Theoretically, when species come to overlap geographically and compete for the same, limited resources, either competitive exclusion or niche partitioning should occur [132,133]. It is possible that the ecological and behavioural differences of *A. mellifera* and *A. cerana* will result in sufficient niche partitioning that both species can co-occur successfully, as was shown in one study in India [134]. It is also possible that both species can coexist if resources, such as flowers, are not limited. 

The two most important resources for cavity-nesting honeybees are floral resources and nest cavities [24]. Competition for pollen and nectar may occur on flowers, or honeybees can attempt to rob honey from other nests (of the same of a different species). Both of these will be examined in turn.

### 10.2. Competition for Floral Resources

Research in Nepal and India found that significantly more *A. cerana* foraged on flowers of different crops, and spent more time on each flower, when *A. mellifera* were absent (Partap, 1998 in [134,135,136]), indicating that *A. mellifera* was the superior competitor. Similar results were found when both species were competing for the same sugar feeding station—*A. mellifera* were more aggressive and successfully and consistently excluded *A. cerana* (Dhaliwal & Atwal, 1970 in [135,137]). *A. cerana* was also found to forage on a number of plant species that *A. mellifera* did not visit, and *vice versa*, and on those plant species that were visited by both species they avoided foraging in the presence of the other species [138].

Two species may also avoid competition if foraging times differ (e.g., *A. cerana* and *A. mellifera* in India; [120]), or if foraging is partitioned spatially (e.g., foraging at top, middle or bottom of trees; *A. cerana vs.* *A. koschevnikovi*, Borneo; [139]). *A. cerana* had a much higher metabolic rate and foragers made many more trips within the same habitat than other species. Foragers also began foraging earlier in the day and they tolerated lower temperatures than *A. mellifera* [66,140]. *A. cerana* are also said to be more industrious while collecting pollen from scattered flowers of a variety of plant species, spending less time on each flower, whereas *A. mellifera* prefer big flower patches of fewer species where they spend more time on each flower (Kuang & Kuang, 2002 in [1,27,140,141]).

Thus, these differences in timing and flower patch preferences may be enough to avoid competitive exclusion. However, further research is needed to confirm this.

### 10.3. Robbing and Direct Fighting

Robbing bees enter another colony’s nest, kill bees and take their honey stores. The smaller the colony the more susceptible it is to robbing [27]. Robbing usually occurs only when floral resources are low, when the nectar flow is interrupted, or when a colony is weak and/or diseased (reviewed in 135]).

Interestingly, *A. mellifera* showed a much stronger defence-response to non-nest mates (of the same or different species) than any of the Asian honeybees examined, which means that *A. mellifera* defended their nest much more strongly than *A. cerana* did [142]. Studies on robbing behaviour between managed hives of *A. mellifera* and *A. cerana* kept at the same apiary showed that although *A. cerana* initiated robbing during lean times, *A. mellifera* usually won, killing the *A. cerana* colony and taking over their foraging area Yang, 2001 in [135,137]. In Japan, robbing of *A. cerana* hives by *A. mellifera* is much more common than robbing of *A. mellifera* hives by *A. cerana* [137]. *A. cerana* were reported to have a very weak defence against intruders and were even observed to feed robber bees [1,137].

Research in Japan on mixed colonies of, and competition between, *A. mellifera ligustica* and *A. cerana*, found that *A. mellifera* behaved much more aggressively towards *A. cerana*, and when placed in confinement together A*. mellifera* were stronger and the superior fighter to *A. cerana* [137]*.* However, *A. cerana* were reported to deliver a powerful bite. When competing for a sugar syrup station, *A. cerana* always lost [137].

However, *A. cerana* appeared to be superior robbers of *A. mellifera* hives on the Solomon Islands [2,143]. In the presence of *A. cerana* and *A. dorsata*, *A. mellifera* also did not thrive in a forest ecosystem on the Philippines [144]. In addition, there was some anecdotal evidence of single occurrences of *A. cerana* robbing Australian native insects (sugar ants, *Camponotus* sp*.*, and a stingless bee, *Trigona* sp.) [29].

Overall, evidence appears to suggest that *A. cerana* is a weak nest defender and competitor compared to *A. mellifera*. If so, it would greatly diminish the treat of *A. cerana* competing with and affecting commercial *A. mellifera*. In fact, recent evidence from North-Eastern Australia seems to support this notion, as *A. cerana* appeared to be outcompeted by *A. mellifera* [79]. Further research into levels of competition between the introduced *Apis* species, as well as between *A. cerana* and Australian native bees will be of utmost importance to determine *A. cerana*’s potential invasiveness.

### 10.4. Mating Interference

It has been observed that *A. mellifera* drones are attracted to *A. cerana* queens and *vice versa*, and that *A. mellifera* drones outcompete *A. cerana* drones when mating with *A. cerana* queens, with detrimental effects on *A. cerana* queens (Ruttner & Maul 1983 in [1,48]). In Pakistan, in the presence of *A. mellifera* drones in an area, virgin *A. cerana* queens did not lay at all or became drone layers [47]. *A. cerana* queens in an *A. mellifera*-dominated area were found to have very low success rate at mating with their own species, especially when *A. mellifera* colonies were in close proximity [1,48]. Similarly, *A. mellifera* queens in an *A. cerana*-dominated area also had very low success rate at mating with their own species [1,48].

Thus, if such mating interference should occur in Australia, it may result in reduced *A. cerana* reproductive success, which in turn would reduce its overall competitiveness and invasiveness. Further research is needed to shed light into this matter.

## 11. Examples where *A. cerana* and *A. mellifera* Co-Exist (through Human Introduction)

### 11.1. Solomon Islands

*A. cerana* and *A. mellifera* did not co-exist successfully on the Solomon Islands, where managed *A. mellifera* declined severely and honey production ceased entirely after *A. cerana* were introduced to the islands in 2003 [2,3]. By 2008, the number of *A. mellifera* hives on Guadalcanal (the main island) had declined from 2000 to five. Although initially it was thought that the introduction of parasitic *Varroa* mites on introduced *A. cerana* caused the decline, this was found not to be the case. Introduced *Varroa* mites were *V. jacobsoni*, which do not breed on *A. mellifera* brood [4]. Losses of *A. mellifera* were attributed to competition for floral resources, *A. cerana* robbing *A. mellifera* hives, leading to starvation in *A. mellifera*, as well as the introduction of the microsporidian pathogen *Nosema ceranae* [2,3,143].

### 11.2. Asia

*A. cerana* and *A. mellifera* co-exist across Asia. *A. cerana* and *A. mellifera ligustica* were kept successfully in close proximity to one another within an apiary in Cambodia [145]. In Pakistan, *A. cerana*, *A. mellifera*, *A. dorsata*, and *A. florea* co-exist, and both *A. cerana* and *A. mellifera* are kept in hives, although reproductive interference occurs between the species [48].

In some areas across Asia where *A. mellifera* have been introduced, widespread declines of *A. cerana* have been observed, particularly in Taiwan, Japan, and China [1,135,137,146,147,148]. Such declines have been attributed to *A. mellifera* being a more aggressive competitor and prone to robbing *A. cerana* nests, leaving *A. cerana* to starve or abscond [149].

Similarly, in Vietnam, *A. mellifera* and *A. cerana* are generally kept in different areas—*A. cerana* do well in the coastal coconut plantations, whereas *A. mellifera* are kept at higher altitudes. However, when both species are brought together in lean times, they were reported to fight, with *A. mellifera* killing or chasing away *A. cerana* without exception [150].

### 11.3. Far North Queensland, Australia

*A. cerana* and *A. mellifera* have been co-existing in the Cairns area of Queensland, Australia, since *A. cerana* was introduced in 2007. No reports of direct robbing attempts on *A. mellifera* hives could be found. Similarly, the impact of *A. cerana* on the local honey production has not been documented. The only observation of *A. cerana* robbing behaviour has been when *A. cerana* robbed wax and honey from an individual “sticky frame” (a single, separated hive frame that has had the honey removed from it) with no *A. mellifera* present (Damon, pers. com.).

However, robbing and taking-over of weakened *A. cerana* nests by *A. mellifera*, as well as strong and effective nest entrance defence by *A. mellifera*, have been observed on several occasions (pers. obs.) as well as by local beekeepers (Damon, pers. com.).

## 12. Control Strategies

In most areas that *A. cerana* is found, it is a native species and so not considered a pest. Therefore, no control strategies have been developed or are necessary for areas where *A. cerana* is endemic. Surveillance is similarly unnecessary in its native range, except perhaps for monitoring its decline and for conservation purposes. Surveillance and control of *A. cerana* as a pest species is mostly conducted in countries where it has been introduced, *i.e.*, New Guinea, the Solomon Islands and Australia. Therefore, the following section is restricted mostly to control strategies in those countries.

*A. cerana* are, to date, only a pest species in Australia, New Guinea, and the Solomon Islands. The majority of literature found covering the detection and control of *A. cerana* was produced by Biosecurity Queensland DAFF, Australia. Very few other control strategies were found documented in the literature for other countries. No control strategies were found for New Guinea, and only three research reports were found for the Solomon Islands: [2,3,143]. The following section will provide a summary of these documents. 

### 12.1. Australia

The Biosecurity Queensland DAFF Asian honeybee program has been in place to control *A.* *cerana* in Queensland, Australia, since its first incursion into Cairns in 2007. Various surveillance and destruction methods were trialled by Biosecurity Queensland DAFF in the past with varying success. Current response measures include passive surveillance (detection and reporting of *A. cerana* nests and swarms by the public), as well as active surveillance in the form of general and targeted floral observations, feeding stations and traps (both offering sugar syrup), and collecting regurgitated crop-pellets from Rainbow bee-eaters (*Merops ornatus*) that are then checked for the presence of *A. cerana* hind wings [12,151,152]. Current destruction methods include the use of aerosol insecticidal spray (Biosecurity Queensland DAFF). Remote treatment of *A. cerana* nests using the broad-spectrum insecticide fipronil has also been trialled, finding low success rates in effectively killing colonies remotely [89].

### 12.2. Solomon Islands

On the Solomon Islands, remote poisoning of *A. cerana* nests using fipronil has been shown to effectively suppress *A. cerana* in a half-square kilometre area for approximately four to eight months [3,143]. The strategy was introduced to local *A. mellifera* beekeepers and involved (a) removing all *A. mellifera* hives to a distance of at least 5 km, (b) training *A. cerana* onto several sugar syrup stations (500 m apart) over a two-week period, (c) remote poisoning *A. cerana* for one hour per feeding station (using 0.05% fipronil solution), and (d) returning the *A. mellifera* hives after a period of four to six weeks [3,143]. This method was not deemed suitable for eradication.

## 13. Detection and Capturing Techniques

### 13.1. Detection

Detection of a newly introduced species is often difficult due to the generally low density of the species in the invaded area [153,154]. Detection of an introduced species at the range boundaries can be even more difficult as populations are often very variable in time and space and at even lower densities [155]. Although *A. cerana* has been found in Australia since 2007 and, thus, is not newly introduced, its current population density would have been affected by the efforts of the Biosecurity Queensland DAFF eradication program between 2007 and 2011.

Detection of *A. cerana* in Australia appears to be difficult, with the most reliable detection methods being public reporting and floral observations [156]. However, these methods are of limited use in dense rainforest or mangroves and remote bushland as floral sources are high up in the trees and human habitation is very low or non-existent. However, early detection of newly introduced species as well as detection at the range boundaries of such species is crucial, and so effective sampling methods need to be developed [153,154]. 

Following is a review of *A. cerana-*specific capture methods used in Asia as well as in Australia and the Solomon Islands.

### 13.2. Swarm Capture

In its native range, *A. cerana* swarms have been captured in order to keep them in hives for thousands of years [7]. Traditional methods of swarm capture generally involve a log hive spread inside with honey and/or wax, which is then hung from a tree—year-round in tropical areas or in spring when scouts are observed looking for nests in temperate areas (e.g., Vietnam, Thailand, Burma, India, Indonesia, Malaysia) [7,157]. In some areas, scout bees are caught and kept inside a log hive for a few days. Once released, they tend to fly back to their swarm and guide it to the log hive [7]. Another method of enticing a swarm into a hive involves tethering or caging a queen inside an empty hive (e.g., after manually removing a queen from her brood comb, or in some areas of India beekeepers sift through swarms with their fingers to find the queen) [7].

In Japan, the orchid *Dendrobium floribunda* (previously *D. pumilum*) attracts drones and foragers [158,159,160], and so the orchid is used to lure swarms to a bait hive.

As part of the eradication program conducted by Biosecurity Queensland, DAFF, *A. cerana* swarms were also captured in order to destroy the colony and prevent the species from spreading. However, swarm traps (made from coconut palm logs) used in Cairns, Australia, were unsuccessful [12].

### 13.3. Trap Attractants—Scents

Honeybees can be trained to recognise scents and identify them with a reward. Thus, scent lures can be used to attract honeybees, but they are not particularly suitable to attract untrained, wild bees. A study on scent preferences in *A. cerana indica* and several strains of *A. mellifera* showed differences between *A. cerana* and *A. mellifera*, as well as slight differences between the different *A. mellifera* strains [161]. Lavender was highly preferred by both *A. cerana* and *A. mellifera*. Scents preferred by *A. cerana* while disliked by *A. mellifera* included orange, jasmine, fennel, and thyme [161].

In the Solomon Islands, the most effective lure was an open flat dish filled with un-scented sugar-syrup placed in sunlight [3]. Offering this lure in the middle of the day seemed to increase bee visitations and reduce visitation by non-target species [3]. Other lures trialled involving scents included acetic acid, isobutanol (odour of molasses), and a mix of citral and geraniol (flower odours), none of which attracted *A. cerana* more than simple sugar-syrup [3].

Further research is required if a scent lure is to be used to attract *A. cerana.*

### 13.4. Trap Attractants—Pheromones

#### 13.4.1. Pheromones in *A. cerana*

Pheromones have been used extensively to lure and trap insects, for example to attract and trap *A. mellifera* swarms e.g., [162]. Unlike scents that need to be learnt and associated with a food reward, pheromones do not need to be learnt—attraction or repulsion to pheromones is inherent but may depend on the concentration. Pheromones that may help attract workers or swarms include queen mandibular gland pheromones, sting apparatus and venom pheromones, as well as homing/orientation pheromones (e.g., Nasonov pheromones). Developing a pheromone attractant for *A. cerana* would be useful in controlling this species where it is unwanted. However, only few studies have been conducted on *A. cerana* pheromones in order to develop a lure.

One such study compared the mandibular gland pheromones of *A. mellifera* and four Asian honeybee species, including *A. cerana* (sourced from Kuala Lumpur, Malaysia, presumably Indo-Malayan *A. cerana*; and from Sri Lanka, India, presumably *A. cerana indica*) [163]. Pheromones of the cavity-nesting species *A. mellifera* and *A. cerana* were more similar to each other than to the open-nesting species, but distinct differences could be found between *A. mellifera* and *A. cerana*. In particular, they shared all but one component—one of the aromatic components, HVA (4-hydroxy-3-methoxyphenylethanol), was absent in *A. cerana.* HVA and ODA ((*E*)-9-oxodec-2-enoic acid) attract a worker retinue around the queen in *A. mellifera* [163]. In addition, relative quantities of each component were different between the species [163].

*A. cerana* Nasonov and queen mandibular pheromones were studied in the Torres Strait islands, Australia [164]. Chemical analysis of two *A. cerana* queens showed similar results to the previous study, including the absence of HVA, but also found a new compound (4-hydroxy-3-methoxyphenyl ethanone) that has not been found in *A. cerana* in any previous study. This result was confirmed in 1999 findings with a further six queens from Java, Indonesia [165].

The most comprehensive study [166] compared pheromone characteristics of *A. cerana* (presumably Indo-Malayan) and *A. nigrocincta* in Sulawesi, Indonesia. Results confirmed a previous study [163]. However, highly different quantities of the different compounds were found, as well as twelve additional compounds in *A. cerana* queens, none of which included the new compound found in [165]. This was attributed the difference in quantities in *A. cerana* to high geographical variation within this species [166].

Eicosenol is an oil and pheromone found in large quantities in *A. cerana* venom [167]. Eicosenol is also found in *A. mellifera*, although not in the venom itself, and at much lower quantities. This compound can be highly attractive to *A. mellifera* workers (Free 1982 in [167]), although mixed results of its attractiveness were found in other studies [167]. Although its function in *A. cerana* is unknown, one suggestion was that Eicosenol may be used by workers to mark particularly rich floral resources so that others can locate those [167]. The use of small quantities of Eicosenol was found to slightly increase the attractiveness of sugar syrup trays to *A. cerana* on the Solomon islands [168]. However, this was not rigorously tested.

Orientation pheromones are exuded by bees at the entrance of their nest from the Nasonov gland on the abdomen. It is also responsible for individual bees staying in a swarm and forming a cohesive unit [169]. Nasonov compounds differ greatly between *A. cerana* (main constituents: linalool, linalool oxide, and citral) and *A. mellifera* (main constituent: geraniol) [164,170,171].

#### 13.4.2. Attractiveness of Pheromones

A mix of the five mandibular gland compounds was found to attract *A. cerana* workers—the sixth component (HVA) found in *A. mellifera* was not required to elicit a full response in *A. cerana* [163].

Kuang *et al.* [172] trialled an *A. cerana* queen pheromone blend (20 mg on a dummy queen), which succeeded in attracting worker bees within a hive (close-range) and also suppressed egg-laying by workers, the building of queen cells, and maintained general colony function.

In a different study, *A. cerana* pheromone experiments were conducted in Papua New Guinea and Java, Indonesia [164,165]. Several pheromones were tested, including a synthetic Indo-Malayan *A. cerana* queen pheromone blend, *A. mellifera* queen pheromone, Nasonov pheromone, and three pheromones present in the sting/venom (Eicosenol, 2-octenyl acetate, and Z-9-octadecenoate). It was found that the synthetic *A. cerana* queen pheromone blend successfully attracted workers at close-range (15 cm) and medium-range (2 m). Preliminary long-range field trials in the Torres Strait showed some success in catching *A. cerana* [165]. However, the trial was limited to three traps. 

Unfortunately, Lacey [164,165] did not elaborate on which components were used, and at what quantities, for the *A. cerana* queen pheromone blend. Therefore, the constituent compounds cannot be compared to results found in other studies e.g., [163,166], which would give an indication of geographic variability in this species.

A range of pheromones were also trialled on the Solomon Islands [3] and on the Torres Strait islands (Shield, J., pers. com.). Unfortunately, sample sizes of both these studies were too low to show any statistically significant results [3].

No experimental studies on pheromone components or attractiveness have been conducted on *A. cerana* in Queensland, Australia.

## 14. Conclusions

The literature review aimed to review the critical points of current knowledge about *A. cerana* in general and tropical *A. cerana* in particular, compare *A. cerana* and *A. mellifera* behaviour and ecology, review *A. cerana* control measures both overseas and in Australia, and highlight gaps in currently available literature and future research needs.

It is apparent how little knowledge is available on tropical *A. cerana* ecology and behaviour in general, and no peer-reviewed scientific publications are as yet available on *A. cerana* in Australia. Most information available on Australian *A. cerana* is in the form of Government reports and unpublished data, which may not be freely available to the scientific community, or indeed anyone wishing to control *A. cerana* in other countries. This highlights an urgent need to publish and disseminate research findings of *A. cerana* in Australia.

Nevertheless, the literature review highlighted some interesting facts and observations, including the apparent docility of *A. cerana* compared to *A. mellifera*, and the occurrence of mating interference between *A. cerana* and *A. mellifera* that is detrimental to *A. cerana* breeding and has led, in some areas, to the decline of *A. cerana* populations. The near collapse of the introduced *A. mellifera* population on the Solomon Islands appears to be an exception. However, it would be most prudent to continue to monitor the Australian incursion, despite little evidence of negative impacts on *A. mellifera* between 2007 and 2012.

Given the large gaps in knowledge about tropical *A. cerana*, it is of upmost importance to conduct research into the general ecology and behaviour of *A. cerana* in Australia (including, for example, determining foraging ranges and times; determining drone and queen flight times and drone aggregation areas to elucidate potential for mating interference; triggers for and timing of swarming and absconding; swarming distances), *A. cerana* pollination (pollen analysis to determine floral sources; potential of *A. cerana* for crop pollination in Australia), the impact of *A. cerana* on the Australian environment (competition with native invertebrates and vertebrates; competition with *A. mellifera*; impact on pollination and reproduction of Australian native plants and weeds); and species distribution and climatic modelling. Most importantly, any new information about *A. cerana* should be used to elucidate its competitiveness and invasiveness in Australia.
